# Systemic analysis identifying PVT1/DUSP13 axis for microvascular invasion in hepatocellular carcinoma

**DOI:** 10.1002/cam4.5546

**Published:** 2022-12-16

**Authors:** Renyi Su, Huizhong Zhang, Lincheng Zhang, Abdul Rehman Khan, Xuanyu Zhang, Rui Wang, Chuxiao Shao, Xuyong Wei, Xiao Xu

**Affiliations:** ^1^ Institute of Organ Transplantation, Zhejiang University Hangzhou China; ^2^ Key Laboratory of Integrated Oncology and Intelligent Medicine of Zhejiang Province, Department of Hepatobiliary and Pancreatic Surgery, Affiliated Hangzhou First People's Hospital Zhejiang University School of Medicine Hangzhou China; ^3^ Department of Hepatobiliary and Pancreatic Surgery, The First Affiliated Hospital Zhejiang University School of Medicine Hangzhou China; ^4^ Department of Hepatobiliary and Pancreatic Surgery, Affiliated Lishui Hospital Zhejiang University School of Medicine Lishui China; ^5^ Westlake Laboratory of Life Sciences and Biomedicine Hangzhou China

**Keywords:** DUSP13, has‐miR‐1258, hepatocellular carcinoma, microvascular invasion, PVT1

## Abstract

**Background:**

Microvascular invasion (MVI) is an independent detrimental risk factor for tumor recurrence and poor survival in hepatocellular carcinoma (HCC). Competitive endogenous RNA (ceRNA) networks play a pivotal role in the modulation of carcinogenesis and progression among diverse tumor types. However, whether the ceRNA mechanisms are engaged in promoting the MVI process in patients with HCC remains unknown.

**Methods:**

A ceRNA regulatory network was constructed based on RNA‐seq data of patients with HCC from The Cancer Genome Atlas (TCGA) database. In total, 10 hub genes of the ceRNA network were identified using four algorithms: “MCC,” “Degree,” “Betweenness,” and “Stress.” Transcriptional expressions were verified by in situ hybridization using clinical samples. Interactions between ceRNA modules were validated by luciferase reporting assay. Logistic regression analysis, correlation analysis, enrichment analysis, promoter region analysis, methylation analysis, and immune infiltration analysis were performed to further investigate the molecular mechanisms and clinical transformation value.

**Results:**

The ceRNA regulatory network featuring a tumor invasion phenotype consisting of 3 long noncoding RNAs, 3 microRNAs, and 93 mRNAs was constructed using transcriptional data from the TCGA database. Systemic analysis and experimentally validation identified a ceRNA network (PVT1/miR‐1258/DUSP13 axis) characterized by lipid regulatory potential, immune properties, and abnormal methylation states in patients with HCC and MVI. Meanwhile, 28 transcriptional factors were identified as potential promotors of PVT1 with 3 transcriptional factors MXD3, ZNF580, and KDM1A promising as therapeutic targets in patients with HCC and MVI. Furthermore, miR‐1258 was an independent predictor for MVI in patients with HCC.

**Conclusion:**

The PVT1/DUSP13 axis is significantly associated with MVI progression in HCC patients. This study provides new insight into mechanisms related to lipids, immune phenotypes, and abnormal epigenetics in oncology research.

## Graphical abstract

In this study, we identified a PVT1/miR‐1258/DUSP13 ceRNA axis characterized with lipid regulatory potential, immune properties, and abnormal methylation states in patients with HCC and MVI. Furthermore, miR‐1258 was an independent predictor for MVI in patients with HCC.

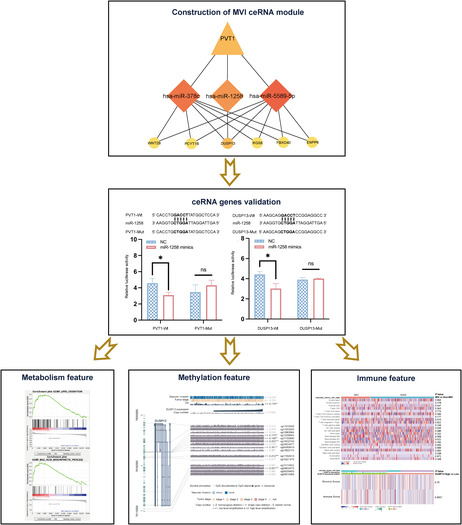

## 1 INTRODUCTION

Liver cancer is the 6th most common cancer worldwide and ranked third for all‐cause cancer deaths in 2020.^1^ The most common primary liver cancer is hepatocellular carcinoma (HCC) which has a poor prognosis with the 5‐year survival rate dropping to 20%—due to the high incidence of metastasis and recurrence.^2^ Microvascular invasion (MVI), a form of micro‐metastasis, has long been recognized as a major independent determinant and a risk factor for tumor recurrence and poor survival after curative liver resection. The efficacy of treatment with curative intent, for example, by radiofrequency ablation, is potentially compromised in the presence of MVI as it reflects an invincible entity for recurrence.^3^ Moreover, a meta‐analysis of 14 studies reported MVI as an independent prognostic factor for overall survival (OS) and disease‐free survival (DFS) in patients with HCC undergoing liver resection.^4^ Although there are certain parameters to predict MVI before resection, diagnosis is almost always based on the pathological specimen. Recently, radiomics have been integrated with imaging techniques to predict MVI in the preoperative clinical setting to encourage the timely adoption of appropriate treatment strategies.^5^, ^6^ The exact pathogenesis of MVI is still an extensive topic of debate and there is a lack of ideal molecular and serum biomarkers to accurately predict its presence.^7^ That said, for precision medicine, it is imperative to use molecular biomarkers to assist imaging in the diagnosis of MVI.

The term “competitive endogenous RNA” (ceRNA) coined by Salmena et al. ushered in a new dawn in physiology and cancer research which proposed to unify the mechanisms that regulate gene expression.^8^ The key argument of the ceRNA hypothesis is that the noncoding RNA (e.g., lncRNA) functions as a microRNA (miRNA) sponge and the RNA transcripts with shared miRNA‐binding sites strive for posttranscriptional control.^9^ Mounting experimental evidence is indicating the ceRNA network is pivotal in modulation as well as the progression of carcinogenesis among diverse tumor types.^10^, ^11^

Regarding the elements of the ceRNA network, the lncRNA–miRNA–mRNA axis is a canonical model in cancer mechanism research.^12^ Xie et al. considered the PDIA3P1–miR‐125/124–TRAF6 ceRNA regulatory axis on chemotherapy in patients with HCC.^13^ Specifically, miR‐125/124 negatively regulates the expression of tumor necrosis factor receptor (TNFR)‐associated factor 6 (TRAF6), whereas PDIA3P1 (an lncRNA) competes with TRAF6 for the miR‐125/124. This last step results in the elevation of TRAF6 expression, which is implicated in the nuclear factor kappa B pathway and thus influences the effects of chemotherapy.

The lncRNA PVT1, located downstream on the human chromosome 8q24, has been implicated in multiple gastrointestinal cancers.^14^ Functional inactivation of PVT1 can alleviate chemoresistance and suppress carcinogenesis and tumor progression.^15^, ^16^ Moreover, the epithelial–mesenchymal transition plays a pivotal role in PVT1‐induced tumor development in prostate cancer cells.^17^ Th DUSP13 gene encodes two isoforms: DUSP13B and DUSP13A.^18^ The DUSP4 and DUSP13 co‐expression promotes transforming growth factor beta‐1‐mediated migration, invasion, and chemoresistance in lung cancer.^19^

Whereas much is known about the ceRNA network in cancer research, the molecular mechanisms of ceRNA in patients with HCC and MVI are limited. In this study, a comprehensive ceRNA network that characterizes carcinogenesis and MVI properties was constructed. Logistic analysis was also adopted to determine independent predictors for MVI in HCC cases. We also performed a systemic analysis to identify the pivotal regulatory networks involved in pathological processes in patients with HCC and MVI. Among the transcription factors (TFs) identified as potential promotors of the ceRNA network, the most promising therapeutic targets to regulate oncogenic activity in HCC cases with MVI were highlighted. This study provides a new mechanistic understanding of patients with HCC and concurrent MVI.

## 2 MATERIALS AND METHODS

### 2.1 Data accession and processing

**FIGURE 1:**
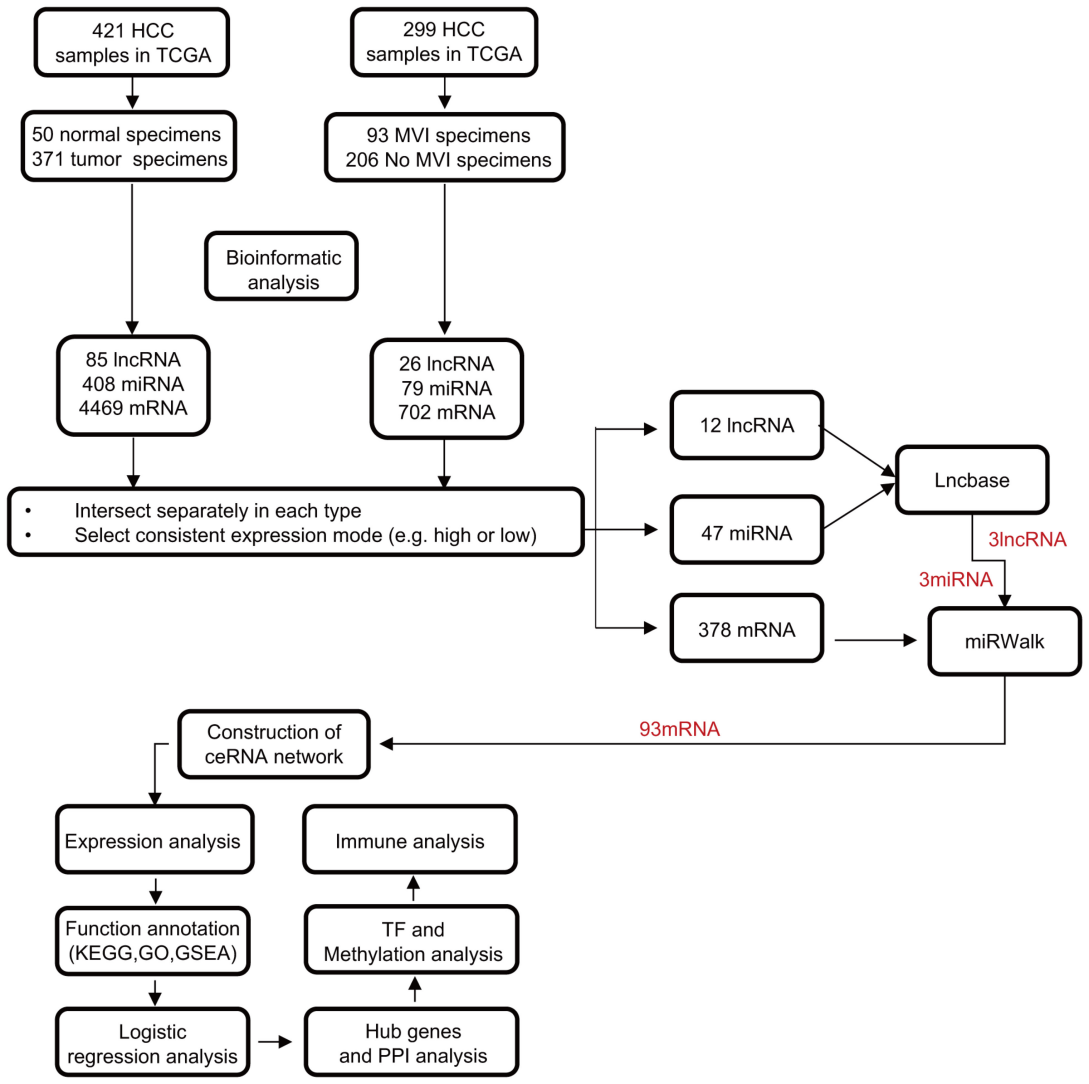
Flowchart of data processing, analysis, and construction of ceRNA regulatory network. HCC, hepatocellular carcinoma; MVI, microvascular invasion.

The flow chart of the study design is shown in Figure 1. The primary data of the patients with HCC were downloaded from the UCSC Xena (https://xenabrowser.net/), which integrated resources from the 141 cohorts and the 1602 data sets facilitating precisive cancer research.^20^ The gene expression (IlluminaHiSeq module), miRNA mature strand expression (IlluminaHiseq module), and clinical information (Phenotypes module) of The Cancer Genome Atlas (TCGA) Liver Cancer cohort were curated from the UCSC Xena website. Gene expression profiles were normalized in the “RSEM” pipeline and log2 was transformed at the UCSC into the Xena repository.^21^ The miRNA expression profiles were added together for isoform with the same miRNA mature strand and log2 was transformed and deposited at the UCSC into the Xena repository. Annotation data for mRNA and lncRNA were downloaded from GENECODE (v19) (https://www.gencodegenes.org/human/) for the subsequent ID conversion using Perl scripts (v5.18.4). ID conversion data for miRNA was downloaded from miRbase (v22) (https://www.mirbase.org/) and transformed using the R programming language (v4.0.3).^22^

### 2.2 Systemically identifying DEGs in HCC

Differential expression analysis was performed in R (v4.0.3) using the EdgeR algorithm to identify differentially expressed genes (DEGs), ‐lncRNAs (DElncRNAs), ‐miRNA (DEmiRNAs), and ‐mRNAs (DEmRNAs).^23^ The univocal criteria adopted for cancer/adjacent normal tissue samples and the MVI/non‐MVI (no microvascular invasion) samples are as follows: (a) |logFC| > 0.3 and *p* < 0.05 as threshold for DElncRNAs; (b) |logFC| > 0.3 and *p* < 0.05 as a threshold for DEmiRNAs; and (c) |logFC| > 1 and *p* < 0.05 as a threshold for DEmRNAs. The volcano plots and the heatmaps of the DEGs were visualized using R (v4.0.3). Names of the top 10 up‐ and downregulated DEGs in |logFC| were displayed with volcano plots.

### 2.3 Construction of ceRNA regulatory network in HCC

The canonical lncRNA–miRNA–mRNA network was based on the ceRNA theory which considers lncRNA as sponges binding miRNA and interacting with mRNA in a competing manner. The construction steps were as follows: (a) to obtain the DEGs that characterize both the carcinogenesis and MVI features, a joint analysis was performed between cancer/adjacent normal tissue samples and the MVI/non‐MVI samples. The DElncRNAs, DEmiRNAs, and DEmRNAs with consistent expression patterns (e.g., upregulation) were obtained after the intersection at each corresponding RNA level for the subsequent ceRNA network construction, (b) to construct the lncRNA–miRNA interactions, DElncRNAs and DEmiRNAs were submitted to the experiments‐based website “LncBase 3.0” (https://diana.e‐ce.uth.gr/lncbasev3/home; performed with default settings) and the prediction module of “LncBase 2.0” (http://carolina.imis.athena‐innovation.gr/diana_tools/web; the threshold was set at 0.7). The lncRNA–miRNA with opposite expression trends were included for the network construction, (c) synthesized results of DEmiRNAs were then uploaded to miRWalk (http://mirwalk.umm.uni‐heidelberg.de/; performed with default settings) to predict the miRNA–mRNA interactions, and (d) herein, a comprehensive lncRNA–miRNA–mRNA regulatory network was constructed by integrating the lncRNA–miRNA and miRNA–mRNA interactions, respectively.

Cytoscape (v3.8.2) software was used to visualize the triple regulatory network. The CytoHubba plugin of Cytoscape was applied to identify the hub gene module of the ceRNA network with the following four algorithms: “MCC,” “Degree,” “Betweenness,” and “Stress.”

### 2.4 Functional annotation analysis

GEPIA (http://gepia.cancer‐pku.cn/) was used to extract the top 100 most similar genes of DUSP13 in HCC cases using the “Similar” button in the “Single Gene Analysis” module. Metascape (http://metascape.org/),^24^ a web tool that provides analysis of functional enrichment, interactive analysis, and gene annotation, was applied to annotate DEmRNAs generated from the lncRNA–miRNA–mRNA regulatory network as well as the DUSP13 and neighboring 100 most similar genes.

Gene Set Enrichment Analyses (GSEA) were conducted using gene sets obtained from the MSigDB database (https://www.gsea‐msigdb.org/gsea/msigdb/index.jsp) and analyzed by the GSEA software (v4.1.0).^25^ The statistical significance of GSEA results was determined by 1000 permutations and default parameters. Gene sets were considered significantly enriched with nominal *p* < 0.05 and normalized enrichment score >1.0.

### 2.5 Localization analysis

The National Center for Biotechnology Information (NCBI) (https://www.ncbi.nlm.nih.gov/) was used to acquire sequences of DElncRNAs. Then, DElncRNAs sequences as query input were uploaded to lncLocator (http://www.csbio.sjtu.edu.cn/bioinf/lncLocator/) to predict subcellular locations.

### 2.6 In situ hybridization analysis

Four tissue microarrays (TMAs) comprising 52 HCC samples and paired adjacent normal tissues were collected from prospective patients undergoing surgical resection at The First Affiliated Hospital, Zhejiang University School of Medicine, China between 2013 and 2016. Details were summarized in Table S1. Another four TMAs were purchased from Shanghai Zhuolibiotech Company Co., Ltd. (Shanghai, China) with 88 patients provided vascular invasion information. Details were summarized in Table S2. The adjacent normal tissues were resected at least 2 cm from the tumor margin and confirmed tumor‐free in the parenchyma biopsy.^26^ The selected specimens were de‐identified, processed, stored, and analyzed as approved by The First Affiliated Hospital, Zhejiang University School of Medicine, China (Reference number: Accelerate approval no. 768). All procedures performed involving human participants were in accordance with the Helsinki declaration and its later amendments or comparable ethical standards.

A total of eight TMAs were established following standard protocols (HK Biotechnology) described previously.^4^ Duplicate 1‐mm cores of the same tissue block were selected for each condition (tumor or adjacent normal tissues) to be included in TMAs. Serial sections (4 μm thick) were coated with 3‐aminopropyltriethoxysilane on slides. TMAs were dewaxed in xylenes and dehydrated through ethanol dilution series. The specimens were digested by proteinase K (20 ug/mL) working solution incubated at 37°C for 20 min. Then, the TMAs were hybridized with PVT1, has‐miR‐1258, has‐miR‐378c, and DUSP13 probe hybridization solution separately (concentration: 500 nM) and incubated in a humidity chamber and hybridized overnight at 42 °C. The signals were detected with a sequence of blocking solution (Rabbit serum), mouse antidigoxigenin‐labeled peroxidase, fresh prepared DAB chromogenic reagent, and hematoxylin staining solution. The results were imaged by using PANNORAMIC (DESK/MIDI/250/1000; 3DHISTECH, Japan). The probe sequences were as follows: PVT1 probe: 5′‐ACAGGGCAGGATCTATGGCATGGGCAGGGTA‐3′; has‐miR‐378c probe: 5′‐CCACTCTTCTGACTCCAAGTCCAGT‐3′; has‐miR‐1258 probe: 5′‐TTCCACGACCTAATCCTAACT‐3′; DUSP13 probe: 5′‐CAGCCGCAGAGGAGAAGTAGGCACTGATGTCAAAA‐3′

A semi‐quantitative scoring method (H‐score) was used for the analysis of in situ hybridization by evaluating the staining intensity and staining percentage of positive cells. It is calculated by the following equation: H score = Ʃ*PC* × *I*, where *I* and *PC* represent the positive intensity (0–3; no = 0 point, weak = 1 point, medium = 2 points, and strong = 3 points) and the percentage of positive cells (0–100), respectively. The final H‐score ranged from 0 to 300. In situ hybridization analyses were performed on pathologically confirmed HCC tissue and paired adjacent normal tissue.

### 2.7 Cell lines and culture

SNU‐449 cells were cultured in Dulbecco's modified Eagle medium (DMEM; Gibco, United States). Further, 10% fetal bovine serum, penicillin (100 units/ml), streptomycin (100 units/ml), L‐glutamine (2 mM), nonessential amino acids, and sodium pyruvate were blended in DMEM and RPMI‐1640. Incubation was performed at 37°C under 5% CO2.

### 2.8 Dual‐luciferase assays

The Duo‐Lite™ Luciferase Assay System (Vazyme Biotech, China) was used for the dual‐luciferase reporter assay. The pmirGLO dual‐luciferase vector was cloned with binding or mutant sequences (Tsingke Biotechnology Co., Ltd., China). Wild‐type or mutant lncRNAs *PVT1* and *DUSP13* were constructed and co‐transfected with has‐miR‐1258 or has‐miR‐378c mimics or negative control (NC), following transfection with jetPRIME (Polyplus) and incubation for 48 h. A microplate reader was used to evaluate the luciferase intensity.

### 2.9 Prediction of transcriptional regulation on PVT1

From the Gene Transcription Regulation Database (GTRD) v20.06 (http://gtrd.biouml.org/),^27^ the chromatin immunoprecipitation (ChIP) data between PVT1 and the TFs were extracted for the subsequent analysis. In the ChIPBase database v2.0 (http://rna.sysu.edu.cn/chipbase/index.php), the stringent selection was performed to extract the TF binding site information related to the promotors.^28^ The binding sites distance was calculated using the following formula:

Binding sites distance = (START + END)/2 – transcription start site (TSS).

The TFs were included and deemed potential promotors for PVT1 in HCC if the following three conditions were met: (a) the shortest binding site distance of TF and TSS was within 1 kbp; (b) experimental ChIP should be performed in HCC‐related cell lines (HepG2 cell line); and (c) Pearson correlation between PVT1 and TFs, miR‐378c and TFs, as well as DUSP13 and TFs in HCC specimens was considered significant with *p* < 0.05.

### 2.10 Methylation analysis

UALCAN (http://ualcan.path.uab.edu/) and Wanderer (http://maplab.imppc.org/wanderer/) were used to explore the DNA methylation state of DUSP13 in cancer and adjacent normal tissue. Moreover, we visualized abnormal methylation sites of DUSP13 in HCC cases using MethSurv (https://biit.cs.ut.ee/methsurv/). Finally, MEXPRESS (https://mexpress.be) was used to explore the relationship between the DUSP13 expression level, the methylation level of CpG islands, and clinical information.

### 2.11 Immune infiltration analysis

To compare immune infiltration between the MVI and non‐MVI groups, the CIBERSORT algorithm was applied to estimate the immune composition of the tumor specimens.^29^ Additionally, multiple immune‐related scores were used to illustrate the immune features between the MVI and non‐MVI groups including the stromal score and immune score. The stromal score and immune score were calculated using the ESTIMATE algorithm.^30^ Furthermore, we explored the correlation of DUSP13 with 22 tumor‐infiltrating immune cells as visualized using R (v4.0.3).

### 2.12 Statistical analysis

The R language software (v4.0.3), GraphPad Prism (v8.0.0), and SPSS Statistics (v26.0.0.2) were used for the statistical analyses. Comparison between groups was performed using a two‐tailed unpaired Student's *t* test or the Wilcoxon rank‐sum test. Correlations were determined using the Pearson correlation coefficient. The logistic regression model was done using SPSS. Adjusted *p* value was adopted for functional enrichment analyses and methylation site analyses. *p* < 0.05 was considered as a statistically significant difference for other statistical analyses. The data processing was mainly based on the “tidyverse” R package while visualized on the “ggplots2” and “pheatmap” R packages.

## 3 RESULTS

### 3.1 Module detection to identify the lncRNA‐, miRNA‐, and mRNA‐specific DEGs

Since MVI is considered an independent risk factor for tumor recurrence and poor prognosis,^3^ we performed a survival analysis based on the TCGA dataset. We found that patients with MVI had a significantly shorter relapse‐free survival than their counterparts without MVI (log‐rank *p* = 0.03; Figure S1).

**FIGURE 2:**
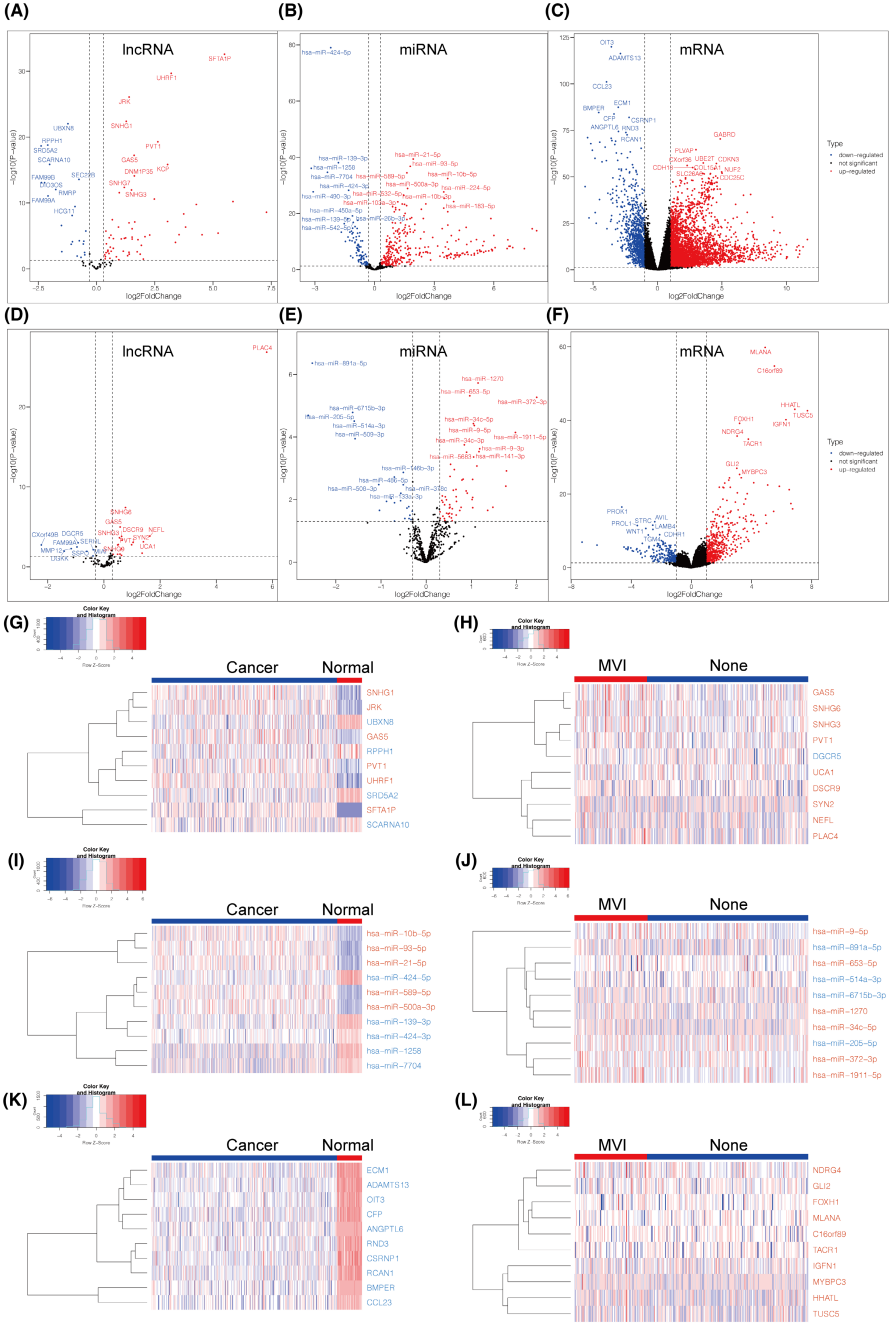
Contrasting the effects of MVI and non‐MVI group on differential gene expression at lncRNA, miRNA, and mRNA levels in HCC. Volcano plots illustrate DEGs of lncRNA, miRNA, and mRNA with the thresholds of |log FC| >0.3, |log FC| >0.3, and |log FC| >1, separately. *p* value <0.05 is considered statistically significant. Volcano plots show DElncRNAs (A), DEmiRNAs (B), and DEmRNAs (C) in cancer/adjacent normal tissue samples and DElncRNAs (D), DEmiRNAs (E), and DEmRNAs (F) in the MVI/ non‐MVI HCC samples. Heatmaps describe DElncRNAs (G), DEmiRNAs (I), and DEmRNAs (K) in cancer/adjacent normal tissue samples and DElncRNAs (H), DEmiRNAs (J), and DEmRNAs (L) in MVI/non‐MVI HCC specimens with samples as horizon axis and top 10 significant DEGs as the vertical axis. DEG, differentially expressed genes; FC, fold change; HCC, hepatocellular carcinoma; MVI, microvascular invasion.

To obtain the DEGs that characterize both carcinogenesis and MVI features, joint analysis was performed on cancer/adjacent normal tissue samples as well as the MVI/non‐MVI samples. As a result, we identified 85 DElncRNAs (62 up‐ and 23 downregulated), 408 DEmiRNAs (312 up‐ and 96 downregulated), and 4469 DEmRNAs (3484 up‐ and 985 downregulated) in the HCC/adjacent normal tissue samples. Moreover, we identified 26 DElncRNAs (18 up‐ and 8 downregulated), 79 DEmiRNAs (56 up‐ and 23 downregulated), and 702 DEmRNAs (490 up‐ and 212 downregulated) in the MVI/non‐MVI HCC samples. Volcano plots indicated DEGs at the transcriptional level from the HCC/adjacent normal tissue samples (Figure 2A–C) and the MVI/non‐MVI HCC samples (Figure 2D–F). The transcriptional heatmaps of the top 10 DEGs with the highest |logFC| were displayed for the HCC/adjacent normal tissue samples (Figure 2G, I, K) and the MVI/non‐MVI HCC samples (Figure 2H, J, L).

### 3.2 Construction and functional annotation of the lncRNA–miRNA–mRNA regulatory network

**FIGURE 3:**
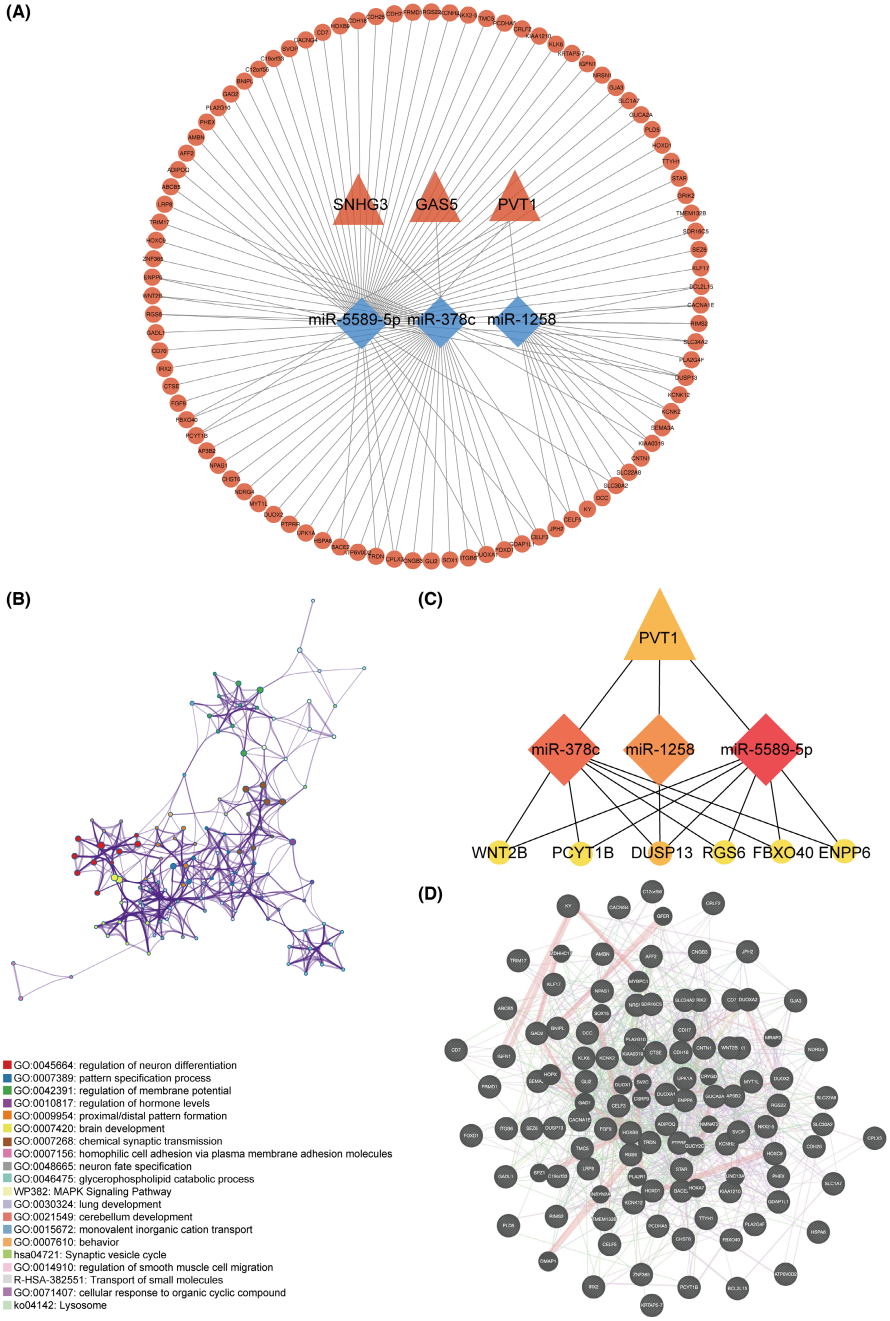
Construction of ceRNA network, hub gene identification, and enrichment analysis. (A) The ceRNA regulatory network in HCC. Triangle = lncRNA; diamond = miRNA; circle = mRNA. Red indicates upregulation, whereas blue indicates downregulation. (B) Functional annotation analysis of 93 DEmRNAs of ceRNA network. (C) Ten hub genes of ceRNA identified using “MCC,” “Degree,” “Betweenness,” and “Stress” algorithms. Triangle = lncRNA; diamond = miRNA; circle = mRNA. (D) Protein–protein interaction map of 93 DEmRNAs at protein levels. Adjusted *p* value <0.05 was considered statistically significant for GO functional enrichment. GO, gene ontology.

The lncRNA–miRNA–mRNA regulatory network was constructed based on the joint analysis of the transcriptional data from the MVI/non‐MVI HCC samples and the HCC/adjacent normal tissue samples. First, intersections were performed for the MVI/non‐MVI HCC samples and HCC/adjacent normal tissue samples at lncRNA, miRNA, and mRNA levels, respectively. To obtain the DEGs characterized by both carcinogenesis and MVI features, DEGs with consistent expression patterns (e.g., upregulation) were selected for the subsequent ceRNA network construction at lncRNA, miRNA, and mRNA levels, respectively. Then 12 DElncRNAs and 47 DEmiRNAs were submitted as input into the LncBase web tool to explore the interactions between the lncRNAs and the miRNAs. Based on the ceRNA hypothesis, lncRNA–miRNA with opposite expression trends were included for network construction, yielding a result of 3 DElncRNAs and 3 DEmiRNAs. To build miRNA–mRNA interactions, the remaining 3 DEmiRNAs were uploaded to the miRWalk website. After the intersection with 378 DEmRNAs and adoption of the opposite expression trend within miRNAs and mRNAs, 93 DEmRNAs were identified referring to the 3 DEmiRNAs. Herein, a ceRNA network integrating the lncRNA–miRNA–mRNA axis which featured HCC carcinogenesis and MVI characteristics was constructed based on the 3 DElncRNAs (3 upregulated), 3 DEmiRNAs (3 downregulated), and 93 DEmRNAs (93 upregulated; Figure 3A).

Since mRNA is a relatively downstream target within the ceRNA triple regulatory network, we conducted functional enrichment annotation of the 93 DEmRNAs with the Metascape webtool (Figure 3B). The top 3 gene ontology (GO) terms were enriched in “regulation of neuron differentiation,” “pattern specification process,” and “regulation of membrane potential.” Moreover, we found items related to the tumor invasion phenotype as regulation of membrane potential, proximal/distal pattern formation, homophilic cell adhesion via plasma membrane adhesion molecules, glycerophospholipid catabolic process, MAPK signaling pathway, and regulation of smooth muscle cell migration.

To identify hub genes of the ceRNA network, the four algorithms “MCC,” “Degree,” “Betweenness,” and “Stress” in CytoHubba were adopted to improve credibility. Intriguingly, we found a unanimous module formed by 1 DElncRNA (PVT1), 3 DEmiRNAs (has‐miR‐378c, has‐miR‐1258, and has‐miR‐5589‐5p), and 6 DEmRNAs (WNT2B, PCYT1B, DUSP13, RGS6, FXO40, and ENPP6; Figure 3C).

To further explore the interactive relationship between the 93 DEGs at the protein level, we constructed a protein–protein interaction network (Figure 3D). Details were presented in Table S3. Congruent with the RNA level, we found the protein‐centered annotation was mainly associated with the regulation of membrane potential, chordate embryonic development, and neurotransmitter transport.

Taken together, we constructed a comprehensive ceRNA network featuring HCC carcinogenesis and MVI characteristics, which presented a tumor invasion phenotype.

### 3.3 Identifying the pivotal PVT1/DUSP13 axis involved in the MVI process

To further determine genes with MVI characteristics, we conducted logistic regression analyses between the MVI features and hub genes generated from the ceRNA network using the TCGA database. In the univariable analysis, we found that MVI was significantly correlated with the expression levels of PVT1 (*p* = 0.019), has‐miR‐1258 (*p* = 0.010), has‐miR‐378c (*p* = 0.039), and DUSP13 (*p* = 0.033; Table 1). The multivariable analysis further confirmed that the transcriptional expression level of has‐miR‐1258 could independently distinguish patients with MVI from non‐MVI counterpart (*p* = 0.036; Table 1).

**TABLE 1:** Logistic regression analysis between MVI feature and hub genes of ceRNA network

	Univariable			Multivariable		
	OR	95% CI	*p* value	OR	95% CI	*p* value
PVT1	1.199	1.03–1.397	0.019*	1.122	0.943–1.335	0.193
miR‐1258	0.675	0.501–0.91	0.010*	0.707	0.512–0.977	0.036*
miR‐378c	0.799	0.645–0.989	0.039*	0.825	0.633–1.074	0.153
miR‐5589‐5p	0.911	0.782–1.062	0.233	0.981	0.81–1.187	0.842
PCYT1B	1.064	0.908–1.246	0.443	1.016	0.841–1.227	0.871
RGS6	0.927	0.775–1.109	0.406	0.999	0.813–1.227	0.992
FBXO40	1.004	0.807–1.248	0.972	1.142	0.877–1.487	0.324
DUSP13	1.162	1.012–1.334	0.033*	1.070	0.913–1.254	0.400
WNT2B	0.976	0.819–1.163	0.782	0.913	0.743–1.121	0.385
ENPP6	1.108	0.992–1.238	0.069	1.117	0.992–1.258	0.069

*Note*: *p* value <0.05 is considered statistically significant.

Abbreviations: CI, Confidence interval; MVI, Microvascular invasion; OR, Odds ratio.

* *p* < 0.05.

**FIGURE 4:**
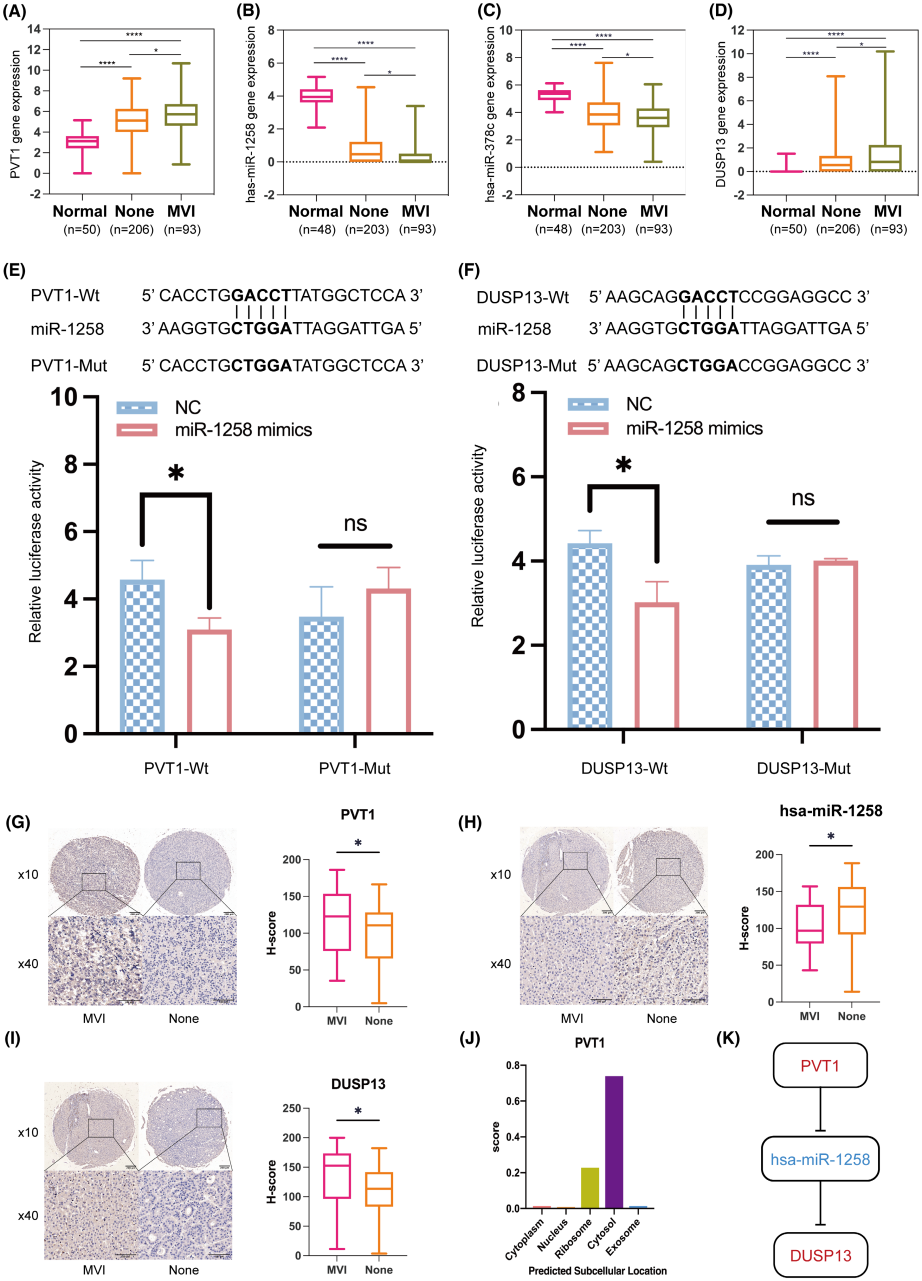
Identification of PVT1/miR‐1258‐miR‐378c/DUSP13 ceRNA axes involved in the MVI process. (A‐D) Expressions of PVT1, miR‐1258, miR‐378c, and DUSP13 separately in HCC cases with MVI or no‐MVI and adjacent normal tissue samples using TCGA data sets. Box range, 25%–75%; whisker range, from minimum to maximum. (E‐F) Luciferase activity of miR‐1258 mimics co‐transfection with PVT1 (E) or DUSP13 (F) determined by dual‐luciferase reporter assay. (G‐I) Quantitative expressions of PVT1, miR‐1258, and DUSP13 separately in MVI or non‐MVI HCC tissue samples by four TMAs. The left panel showed representative images from TMAs of HCC. The left top = HCC with MVI, right top = HCC without MVI. Scale bar =200 μm. Bottom = magnified figures of representative regions for MVI and non‐MVI HCC tissues, separately. Scale bar =100 μm. The right panel showed H‐score between 26 HCC with MVI and 62 HCC without MVI tissues. (J) Subcellular location of PVT1 predicted by lncLocator webtool. (K) Schematic illustration of ceNRA network. PVT1 negatively regulates miR‐1258, whereas miR‐1258 negatively regulates DUSP13. Significance = not significant (ns), ⁎*p* < 0.05, ⁎⁎*p* < 0.01, ⁎⁎⁎*p* < 0.001, ⁎⁎⁎⁎*p* < 0.0001. HCC, hepatocellular carcinoma; TMAs, tissue microarrays.

To investigate the role of PVT1, miR‐378c, miR‐1258, and DUSP13 in the MVI process, we quantitively delineated the discrepancy in transcriptional expression between the MVI group, non‐MVI group, and adjacent normal tissue samples. As expected, results showed a similar escalated expression pattern within PVT1 and DUSP13 but the opposite for miR‐378c (Figure 4A). Specifically, the expression level of PVT1 was significantly higher in the MVI compared with the non‐MVI group (*p* < 0.05) and normal controls (*p* < 0.0001) as well as significantly upregulated in the non‐MVI group compared with normal controls (*p* < 0.0001). A similar trend for DUSP13 was observed that showed a higher expression level of DUSP13 in the MVI than in the non‐MVI group (*p* < 0.05) and normal controls (*p* < 0.0001) as well as higher in the non‐MVI group compared with normal controls (*p* < 0.0001; Figure 4D). Moreover, the expression patterns of miR‐378c and miR‐1258 exhibited a consistently opposite de‐escalation trend (Figure 4B, C). The expression of miR‐378c and miR‐1258 were higher in normal controls compared with the MVI (*p* < 0.0001) and non‐MVI group (*p* < 0.0001) as well as significantly upregulated in the non‐MVI compared with the MVI group (*p* < 0.05).

To corroborate the abnormal expression patterns, in situ hybridization was performed on the four TMAs comprising 52 paired human HCC tissues and adjacent normal tissues (Table S1). Intriguingly, we found congruent significant high expression patterns of PVT1 (Figure S2A) and DUSP13 (Figure S2D) in tumor tissue compared with the normal counterpart. However, despite no robust differences observed between the tumor and normal tissue regarding expression levels of has‐miR‐1258 (Figure S2B) and has‐miR‐378c (Figure S2C), there were similar de‐escalation trends in the tumor tissue, consistent with the aforementioned results. To determine the associations between the identified competing ceRNA modules and MVI, four other TMAs comprising 26 MVI and 62 non‐MVI HCC tissues were used. Consistent with the ceRNA modules from The Cancer Genomic Atlas database, upregulation trends of PVT1 (*p* = 0.044; Figure 4G) and DUSP13 (*p* = 0.018; Figure 4I), downregulation trend of has‐miR‐1258 (*p* = 0.043; Figure 4H), and no significant change in has‐miR‐378c (*p* = 0.083; Figure S2E) was found between MVI and non‐MVI HCC tissues.

To verify the potential ceRNA interactions experimentally, we performed luciferase reporter assays. The results showed that has‐miR‐1258 mimics significantly suppressed wild‐type PVT1 reporter luciferase activity compared with that of NC and wild‐type pmirGLO‐PVT1 co‐transfection (*p* = 0.018; Figure 4E), but not that of the mutant PVT1 (*p* = 0.251; Figure 4 E). Similarly, has‐miR‐1258 mimics significantly suppressed wild‐type DUSP13 reporter luciferase activity compared with that of the NC and wild‐type pmirGLO‐DUSP13 group (*p* = 0.013; Figure 4F) but not mutant DUSP13 (*p* = 0.475; Figure 4F). However, the interaction between has‐miR‐378c and PVT1 (Figure S2F) or has‐miR‐378c and DUSP13 (Figure S2G) was not significant.

To verify the potential ceRNA interactions experimentally, we performed luciferase reporter assays. The results showed that has‐miR‐1258 mimics significantly suppressed PVT1 wild‐type reporter luciferase activity compared with the co‐transfections NC + pmirGLO‐PVT1 wild group (*p* = 0.018; Figure 4E), while no siginificance was observed in PVT1‐mutant group (*p* = 0.251; Figure 4E). Similarly, we found has‐miR‐1258 mimics significantly suppressed DUSP13 wild‐type reporter luciferase activity compared with the co‐transfections NC + pmirGLO‐DUSP13 wild group (*p* = 0.013; Figure 4F), while no siginificance was observed in DUSP13‐mutant group (*p* = 0.475; Figure 4F). However, no statistical significance was observed for interaction between has‐miR‐378c and PVT1 (Figure S2F) or has‐miR‐378c and DUSP13 (Figure S2G).

Given that subcellular location was engaged in determining the function of lncRNA, we used the lncLocator web tool to predict the subcellular location. We found lncRNA PVT1 was primarily enriched in the cytosol that might function in ceRNA mechanisms that sponge miR‐1258 to promote tumor invasion (Figure 4J).

Taken together, the above results with experiment validation provided evidence for PVT1/miR‐1258/DUSP13 axis involving the MVI process in HCC patients (Figure 4K).

### 3.4 Association between DUSP13 and lipid metabolism in the MVI process

**FIGURE 5:**
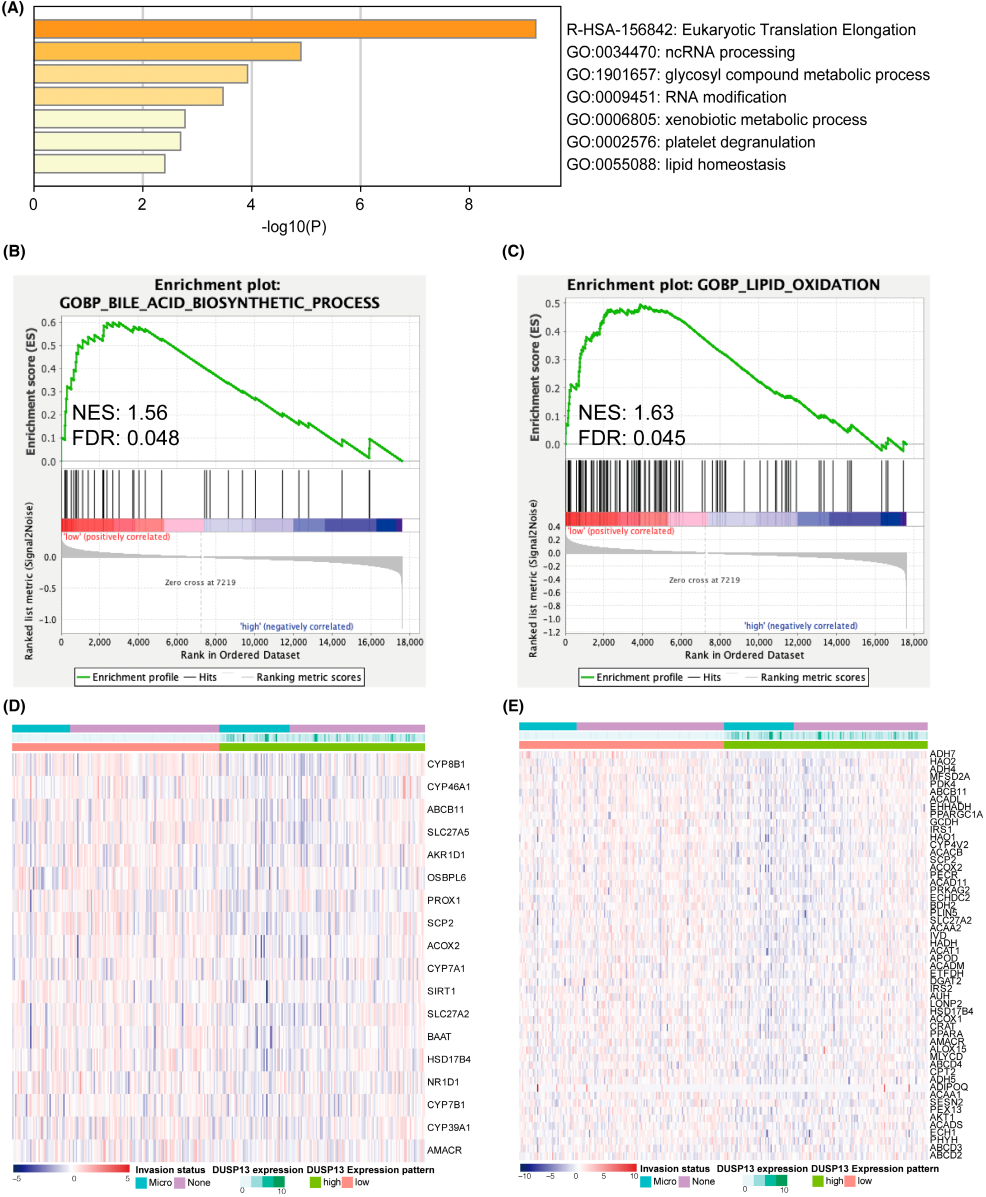
Functional enrichment analysis revealing lipid‐centered feature of PVT1/DUSP13 axis. (A) Functional enrichment of DUSP13 and neighboring 100 similar genes. (B) Correlation between DUSP13 and bile acid biosynthetic process by GSEA analysis. (C) Correlation between DUSP13 and lipid oxidation by GSEA analysis. Adjusted *p* value <0.05 was considered statistically significant for GSEA functional enrichment. (D) Heatmaps of DUSP13 expression levels and genes in bile acid biosynthetic process. (E) Heatmaps of DUSP13 expression levels and genes in lipid oxidation. GSEA, Gene Set Enrichment Analyses.

To further explore the function of DUSP13, enrichment analysis (including GO and KEGG) was performed to annotate mRNA DUSP13 with its neighboring 100 most similar genes predicted by the GEPIA webtool. As shown in Figure 5A, DUSP13 and its neighboring genes were mainly enriched in noncoding RNA processing and diverse metabolic regulation of processes including the glycosyl compound metabolic process, xenobiotic metabolic process, and lipid homeostasis.

Intriguingly, a recent study showed that dysfunction of lipid metabolism was involved in MVI and metastatic transmigration in HCC cases.^31^ Therefore, we hypothesized that the PVT1/DUSP13 axis may present with lipid‐centered regulating manners in patients with HCC and MVI. First, GSEA analysis showed that a significant correlation exists between the bile acid biosynthetic process (FDR = 0.048; Figure 5B) and lipid oxidation (FDR = 0.045; Figure 5C) concerning the expression level of DUSP13.

Moreover, the heatmaps showed highly expressed DUSP13 presented with downregulated patterns of genes involving bile synthesis (Figure 5D) and lipid oxidation (Figure 5E). Taken together, these results indicated that DUSP13 as a downstream target of the PVT1/DUSP13 axis was involved in the modulation of lipid metabolism that might be a potential mechanism facilitating the MVI process in patients with HCC.

### 3.5 Identification of transcription factors for lncRNA PVT1

To identify potential targets for clinical therapeutic intervention at the genetic level, we performed a prediction analysis to investigate potential binding sites of TF on the promotors of PVT1 using GTRD (v20.06) and ChIPBase (v2.0). ChIP data were extracted from GTRD and following ChIP analysis was conducted on the HCC cell line HepG2 to predict the promotors of PVT1 within the upstream 1 kbp from the TSS. Moreover, to maximize the prediction performance for anticipating promotors of PVT1 by modulating the PVT1/DUSP13 axis potential in HCC cases, we conducted co‐expression analysis to identify molecules significantly associated with HCC specimens. For lncRNA PVT1, we identified 28 TFs achieving our preset settings (Table S3). Additionally, the three TFs MXD3, ZNF580, and KDM1A were significantly correlated to PVT1, miR‐378c, miR‐1258, and DUSP13 molecules with moderate correlations (all absolute Pearson correlation coefficients reaching 0.3), which were indicated as promising targets for pharmacological intervention in mechanistic studies.

### 3.6 Clinical relevance of DUSP13 methylation level

**FIGURE 6:**
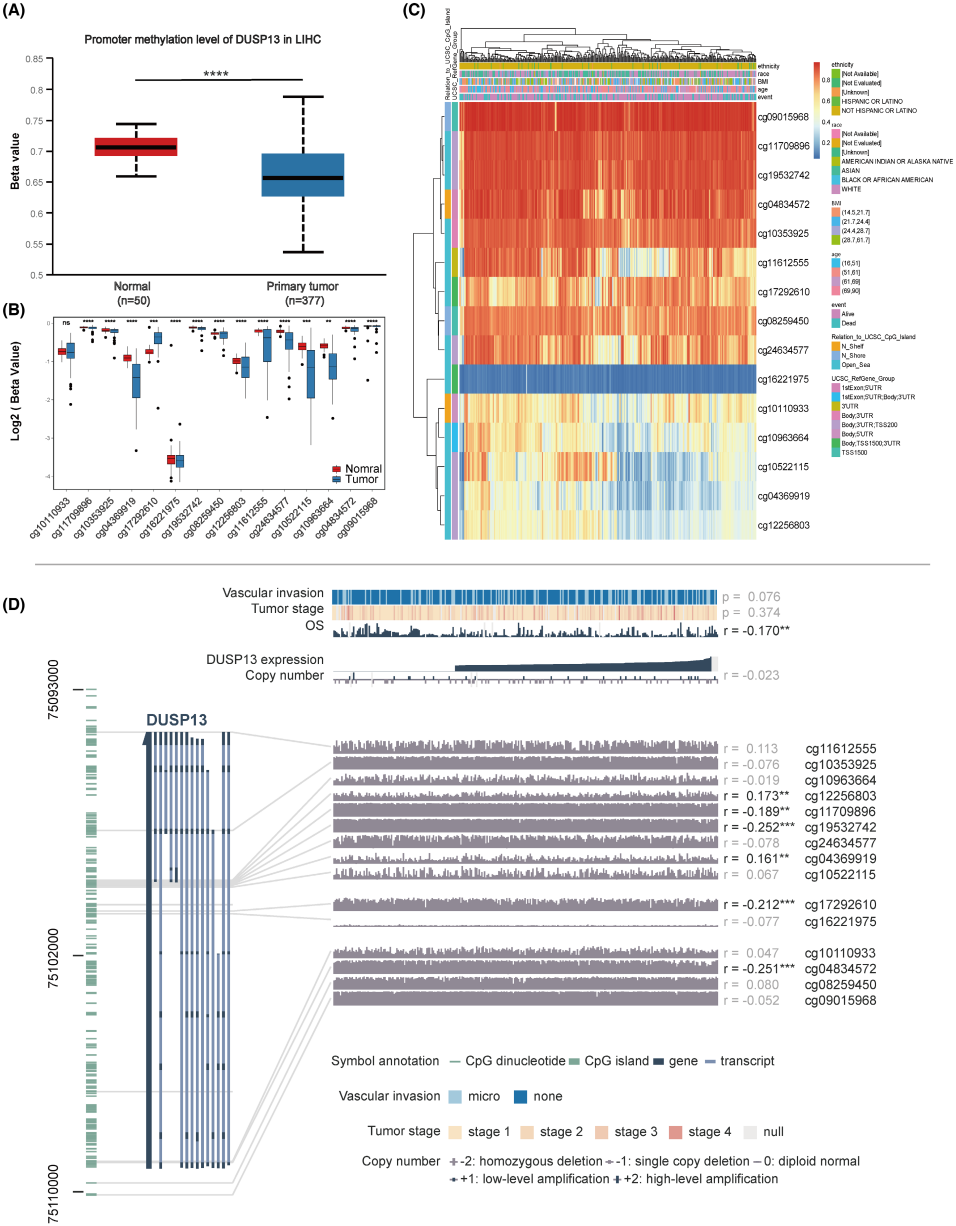
Methylation analysis of DUSP13. (A) The methylation level of DUSP13 was evaluated by UALCAN. (B) The methylation level of DUSP13 with 50 paired tumor and normal tissues using TCGA data set. Adjusted *p* value <0.05 was considered statistically significant. (C) Heatmap of abnormal methylation sites of DUSP13 in HCC cases using MethSurv. (D) Relationship between DUSP13 expression level, methylation level of CpG islands, and clinical information. Methylation sites of DUSP13 were presented in the left panel. DUSP13 expression level and clinical information were presented at the top of the middle panel. P value and/or Pearson's correlation coefficients were presented in the right panel. Significance = ⁎*p* < 0.05, ⁎⁎*p* < 0.01, ⁎⁎⁎*p* < 0.001, ⁎⁎⁎⁎*p* < 0.0001.

To give a thorough elucidation of aberrant expression of DUSP13 in HCC cases, we conducted multiple analyses exploring the potential influence of methylation on DUSP13 expression. First, a relatively low methylation level of DUSP13 was presented in HCC samples compared with normal tissue using UALCAN (*p* < 0.0001, Figure 6A). Moreover, we visualized the methylated sites of DUSP13 with 50 paired tumors and adjacent normal tissues and found a similar low methylation level of DUSP13 in HCC cases in general (adjusted *p* < 0.05, Figure 6B). Furthermore, relative expression levels of methylation sites of DUSP13 were presented in heatmaps by MethSurv. Intriguingly, we found a methylation site cg11612555 located in the open sea and 3′UTR region (Figure 6C). To interrogate the relationship between the methylation status of DUSP13 and the expression level of DUSP13 in HCC specimens, we performed correlation analysis and found four methylation sites (cg11709896, cg19532743, cg17292610, and cg04834572) were significant inversely proportional to DUSP13 expression (Figure 6D). We also showed the expression level of DUSP13 was negatively correlated with OS (*p* < 0.01, Figure 6D).

### 3.7 Comparison of immune infiltration between the MVI and non‐MVI groups

**FIGURE 7:**
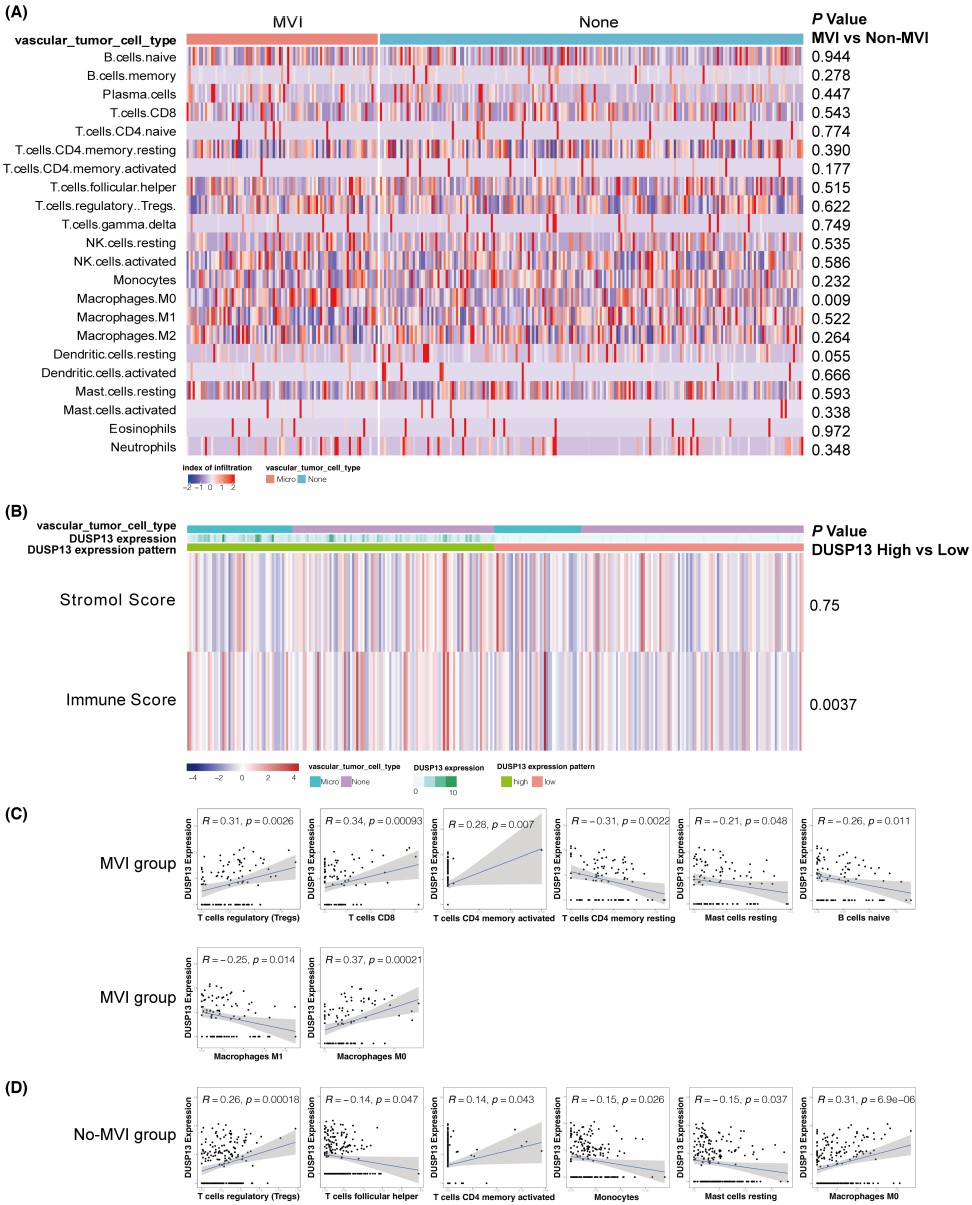
Correlation between DUSP13, immune infiltration, and invasion status in HCC. (A) Heatmaps of immune cells in MVI and non‐MVI groups. The middle panel shows the infiltration index of immune cells. The right panel shows a discrepancy in immune infiltration in MVI and non‐MVI groups. (B). Heatmaps of DUSP13 expression and multiple immune scores. The middle panel shows different immune‐related scores. The right panel shows immune scores in different DUSP13 expression levels. Red indicates upregulation, whereas blue indicates downregulation. (C) Significant correlation of DUSP13 and immune infiltration in MVI group. (D) Significant correlation of DUSP13 and immune infiltration in non‐MVI group. *p* value <0.05 was considered significant. HCC, hepatocellular carcinoma; MVI, microvascular invasion.

Recent studies established a strong connection between the immune infiltration and tumor MVI phenotype in diverse gastrointestinal cancers including HCC.^32^, ^33^, ^34^ Hence, we further characterized the discrepancy of immune features within the MVI and non‐MVI groups in HCC cases. To construct a global tumor immune atlas, we conducted immune cell identification by evaluating marker genes using the CIBERSORT algorithm. The composition ratio analysis showed a shared pattern of immune cell subtypes within both the MVI and non‐MVI counterparts, although with different immune proportions (Figure 7A). The immune infiltration was mainly composed of macrophage and T cell subtypes accounting for over half of the immune cells, which was consistent with previous results.^33^ To further meticulously identify immune cells engaged in the MVI process, we performed a differential analysis of immune cells between the MVI and non‐MVI counterparts. Intriguingly, we found that macrophage M0 was significantly higher in the MVI compared with the non‐MVI group regarding the proportion of immune infiltration (*p* = 0.009; Figure 7A). However, no discrepancy was observed among the MVI and non‐MVI groups regarding classic immunosuppressive T cell regulatory cells (Tregs) (*p* = 0.622; Figure 7A). Additionally, the multiple immune‐related score analysis showed DUSP13 expression level was only positively associated with the immune score (*p* = 0.0037; Figure 7B).

To further investigate the role of DUSP13 in immune infiltration, correlation analysis was performed to compare and identify immune cells that potentially operated on the PVT1/DUSP13 axis in HCC cases with MVI (Figure 7C). We found similar significant correlations of DUSP13 and immune infiltration in macrophage M0 (*p* < 0.001 in Figure 7C; *p* < 0.001in Figure 7D, respectively), Tregs (*p* = 0.0026, Figure 7C; *p* < 0.001, Figure 7D, respectively), activated memory CD4 T cell (*p* = 0.007, Figure 7C; *p* = 0.043, Figure 7D, respectively), and resting mast cells (*p* = 0.048, Figure 7C; *p* = 0.037, Figure 7D, respectively) regarding the MVI and non‐MVI group. However, we found significant correlations of DUSP13 and immune infiltration in CD8 T cell (*p* < 0.001), resting memory CD4 T cell (*p* = 0.007), naïve B cell (*p* = 0.011), and macrophage M1 (*p* = 0.014) regarding the MVI but not in the non‐MVI group, indicating that PVT1/DUSP13 axis may promote the MVI process via interaction with these immune infiltrating cells in HCC.

## 4 DISCUSSION

MVI plays a substantial role in HCC progression and recurrence.^35^, ^36^ In the context of precision medicine, there is a trend to utilize molecular biomarkers in assisting imaging techniques to pre‐operatively diagnose MVI. However, an enormous gap exists between the effective clinical implication of these biomarkers and the underlying molecular mechanisms of MVI in the setting of HCC. Here, we identified a PVT1/DUSP13 ceRNA triple regulatory network featured lipid metabolism and immune phenotypes in patients with HCC and MVI. First, according to the canonical ceRNA hypothesis, we obtained 3 DElncRNAs, 3 DEmiRNAs, and 93 DEmRNAs which feature in HCC carcinogenesis and MVI. The tumor invasion phenotype was corroborated by functional annotations at the RNA and protein levels. Next, 10 hub genes were identified for the ceRNA network by combining four algorithms. Since an axis with clinical translation value is promising for subsequent studies, the potential clinical correlation between the ceRNA network and MVI features was further explored. Herein, we found the PVT1/miR‐378c/DUSP13 and PVT1/miR‐1258/DUSP13 regulatory ceRNA axes involved in the MVI process as confirmed by analysis of transcriptional expression, correlation, and localization.

In HCC cases, great attention has been bestowed to the PVT1‐mediated lncRNA–miRNA–mRNA ceRNA axis.^37^, ^38^ Our transcriptional data identified three hub genes (PVT1, miR‐1258, DUSP13) features within the invasion phenotype. Many of them have reportedly been associated with tumor invasion and metastasis. Preclinical research has shown that the upregulated PVT1 not only promotes cell proliferation, cell cycling, and stemness phenotypes but also facilitates migration, invasion, and cell apoptosis in HCC.^39^, ^40^ Confirmed in vitro and in vivo, miR‐1258 is engaged in a feedback loop consisting of β‐catenin/TCF‐4–LINC01278–miR‐1258–Smad2/3, while downregulated miR‐1258 promotes migration and invasion in HCC cells.^41^ Similarly, our multivariable analysis showed miR‐1258 was an independent predictive factor for MVI. However, more clinical research is warranted to consolidate the credibility of the predictive power of these molecules at the transcriptional level.

Currently, there are limited oncology research focusing on investigating the mechanism of DUSP13.^42^, ^43^ Since genes with similar expression patterns are prone to have related functions,^44^ we performed synergistic GSEA analysis and functional enrichment analysis of the downstream DUSP13 molecule and 100 most similar genes to annotate the identified PVT1/DUSP13 axis. We found DUSP13 was involved in multiple tuning modes of lipid metabolism. GSEA analysis showed that DUSP13 as a downstream target of the PVT1/DUSP13 axis had a significant correlation with the bile acid biosynthesis process and lipid oxidation. This is consistent with previous studies that demonstrated the dysregulation of key enzymes (e.g., cytosolic phospholipase A2α and proliferator‐activated receptor γ) in lipid metabolism could promote MVI in HCC—suggesting a pivotal role for lipid metabolism in HCC progression.^31^, ^45^, ^46^ Additionally, functional annotation analysis revealed that DUSP13 was primarily enriched in metabolic‐centered processes (e.g., glycosyl compound metabolic process and xenobiotic metabolic process), indicating a stupendous potential for the PVT1/DUSP13 axis as a regulatory hub to participate in the modulation of multiple metabolic pathways. To our knowledge, the present study is the first to identify an lncRNA–miRNA–mRNA ceRNA regulatory network featured with modulation of lipid metabolism in patients with HCC and MVI, thus providing new insight for further mechanistic research.

A previous study reported a tight relationship between DNA methylation and DUSP13 in epithelial ovarian cancer.^43^ Hence, in this study, multiple analysis methods were adopted to examine the methylation level of DUSP13 in HCC with MVI. We found a relatively low methylation level of DUSP13 in HCC cases in general. Intriguingly, in the systemic analysis of DUSP13 and the methylation level, DUSP13 expression was significantly correlated with seven methylation sites, among which five sites presented a negative correlation. Since a hypomethylated state of CpG sites generally leads to increased gene expression, these five hypomethylation sites (cg11709896, cg19532743, cg17292610, cg04834572, and cg26105766) may be accountable for aberrant upregulated expression of DUSP13 and potentially promote carcinogenesis.^47^ Of note, the DUSP13 expression level was negatively correlated to OS, which represented a degree of malignancy correlating with the PVT1/DUSP13 axis in HCC with MVI.

Accumulating evidence highlights the interactions between lncRNA and TFs as well as canonical mRNA and TFs at the transcriptional level.^48^, ^49^, ^50^ In the current study, we identified 28 TFs with the 3 TFs MXD3, ZNF580, and KDM1A significantly correlated to the PVT1/DUSP13 axis with moderate correlation (all with absolute Pearson correlation coefficients reaching 0.3) experimentally validated in the HCC cell line HepG2 using the ChIP assay. Since the interplay between the TFs and RNA binding sites tune the downstream expression of the target gene,^51^ the 28 identified TFs for PVT1 may function as potential biomarkers and/or therapeutic targets in the HCC cases.

Erstwhile studies bridged the gap between the immune and tumor MVI phenotype in diverse gastrointestinal cancers including HCC.^32^, ^33^, ^34^ In this study, we found that the composition of the immune cells within the MVI and non‐MVI counterparts shared a generally similar pattern but different immune proportions of macrophages and T‐cell subtypes, which is consistent with previous findings.^33^ A recent study demonstrated the M0 subtype was an independent prognostic factor for OS in HCC.^52^ Furthermore, our results meticulously showed compositions of the M0 subtype in different MVI states, which may explain the worse survival in the MVI group. Largely consistent with previous studies showing the immune infiltration state and immune‐related genes played a pivotal role in HCC with MVI,^53^, ^54^ our data showed that the DUSP13 level was significantly associated with the immune score and immune infiltration by the naïve B cell, CD8 T cell, resting memory CD4 T cell, activated memory CD4 T cell, and macrophage M1 in the MVI but not non‐MVI group, suggesting great tuning potential by immunotherapy. Taken together, these results indicate that the PVT1/DUSP13 axis may promote the MVI process via interaction with immune infiltrating cells in HCC.

The caveat of this study is the use of a single data set from the TCGA and a bioinformatic‐centered analysis strategy with limited experiments. Moreover, our statistical analysis was mainly based on transcriptional data, which did not consider the molecular alternation by other omics levels such as the novel single‐cell level that provides a new perspective to investigate the relationship.^55^, ^56^ Thus, a systemic analysis of omics data is required to evaluate the significance of the PVT1/DUSP13 axis in HCC cases with MVI. Finally, the clinical predictive power of miR‐1258 for MVI should be further validated in large clinical trials. Follow‐up research could also use the plasma of patients as a noninvasive detection method to improve the ease of clinical translation.

In summary, we constructed a comprehensive ceRNA network characterized by a tumor invasion phenotype and identified a PVT1/DUSP13 axis featured with lipid regulatory potential, immune properties, and abnormal methylation states in patients with HCC and MVI. Three TFs (MXD3, ZNF580, and KDM1A) were identified as promising therapeutic targets to modulate the activity of the PVT1/DUSP13 axis in HCC cases. Last, the miRNA has‐miR‐1258 was identified as a potential predictor for HCC with MVI cases, thus providing new insights for researchers and clinicians to explore translational medicine for patients with HCC and concurrent MVI.

AUTHOR CONTRIBUTIONS

RYS was involved in data curation, project administration, resources, formal analysis, visualization, and writing—original draft; HZZ was involved in project administration, investigation, and writing—original draft; LCZ was involved in data curation, project administration; ARK was involved in project administration, resources, and writing—review and editing; XYZ was involved in project administration; RW was involved in methodology, project administration, software, and visualization; CXS was involved in conceptualization and supervision; XYW was involved in conceptualization, project administration, funding acquisition, investigation, review and editing; XX was involved in conceptualization, funding acquisition, investigation, resources, supervision, and writing—review and editing.

## AUTHOR CONTRIBUTIONS

**Renyi Su:** Data curation (equal); formal analysis (equal); project administration (equal); resources (equal); visualization (equal); writing – original draft (equal). **Huizhong Zhang:** Investigation (equal); project administration (equal); writing – original draft (equal). **Lincheng Zhang:** Data curation (equal); project administration (equal). **Abdul Rehman Khan:** Project administration (equal); software (equal); visualization (equal). **Xuanyu Zhang:** Project administration (equal). **Rui Wang:** Methodology (equal); project administration (equal). **Chuxiao Shao:** Conceptualization (equal); supervision (equal). **Xuyong Wei:** Conceptualization (equal); funding acquisition (equal); investigation (equal); project administration (equal); writing – review and editing (equal). **Xiao Xu:** Conceptualization (equal); funding acquisition (equal); investigation (equal); resources (equal); supervision (equal); writing – review and editing (equal).

## CONFLICT OF INTEREST

The authors declare that the research was conducted in the absence of any commercial or financial relationships that could be construed as a potential conflict of interest.

## ETHICS STATEMENT

This study was approved by the Ethics Committee of the First Affiliated Hospital, Zhejiang University School of Medicine. All procedures performed in studies involving human participants were in accordance with the ethical standards of the Ethical Committee at the First Affiliated Hospital, Zhejiang University School of Medicine, and with the 1983 Helsinki declaration and its later amendments or comparable ethical standards. A signed informed consent form was obtained from each patient or their family members, who were provided with a detailed explanation about the study.

## Supporting information


Figure S1.

Figure S2.

Figure S3.

Table S1.

Table S2.

Table S3.

Table S4.

## Data Availability

Publicly available dataset was analyzed in this study. This data can be found in TCGA downloaded from UCSC xena (https://xenabrowser.net/). Other related data are all accessible within this paper.
