# Supplementary figures and images for: Systemic analysis identifying PVT1/DUSP13 axis for microvascular invasion in hepatocellular carcinoma
Source: Cancer Med. 2022 Dec 16;12(7):8937–55. doi: 10.1002/cam4.5546 (PMC10134337; doi:10.1002/cam4.5546)

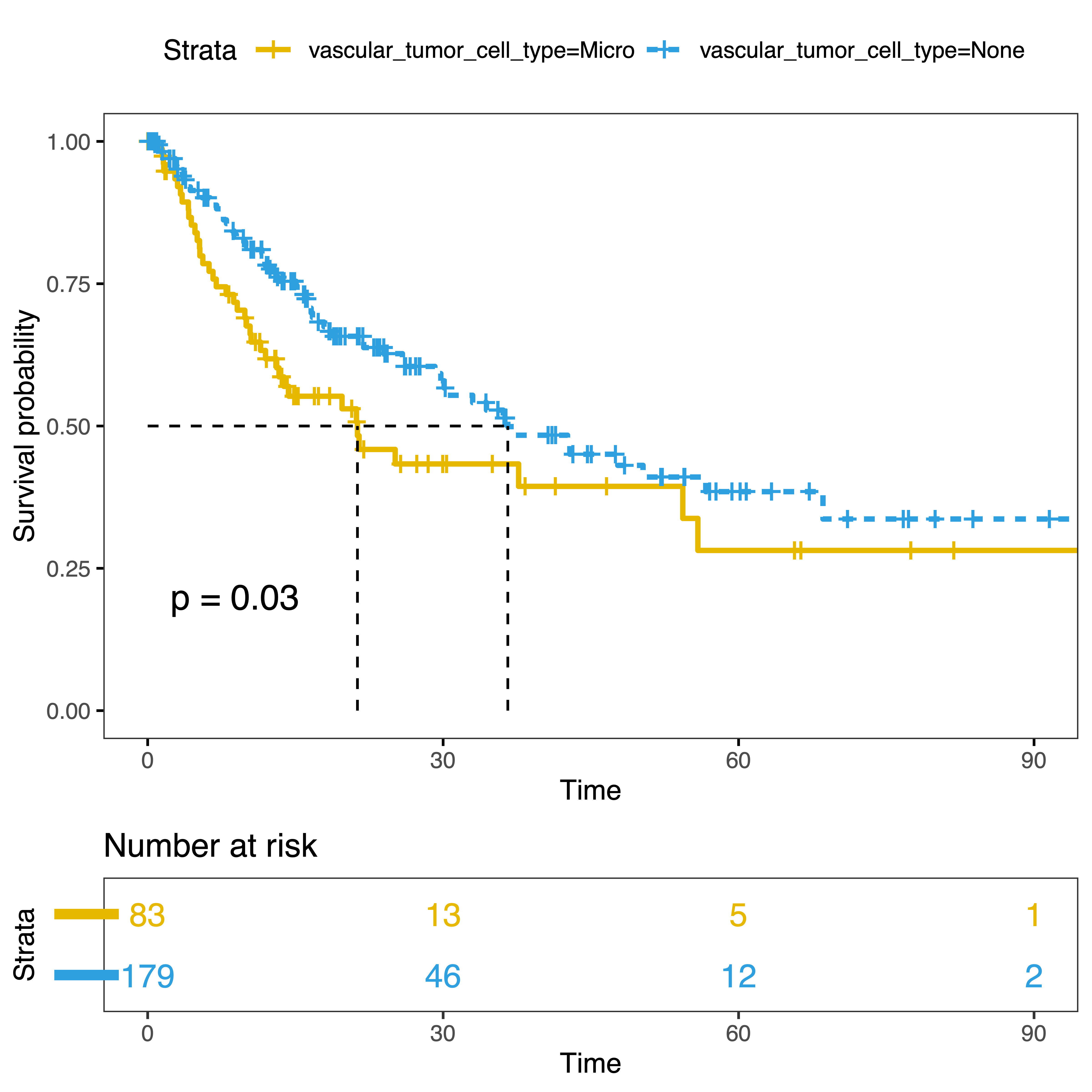

Supplement: Supplementary file 1 — Figure S1. [file CAM4-12-8937-s001.tiff]

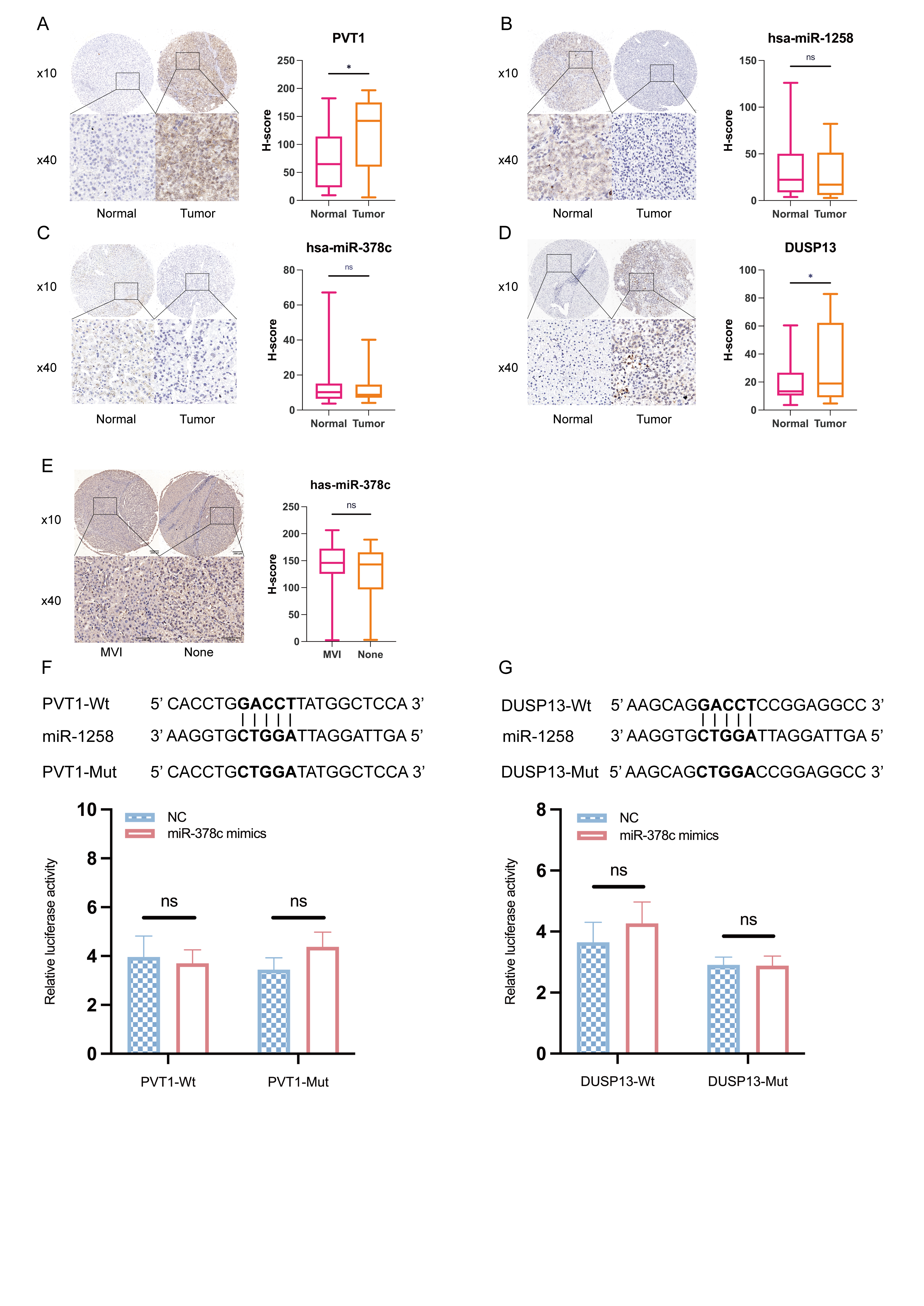

Supplement: Supplementary file 2 — Figure S2. [file CAM4-12-8937-s007.tiff]

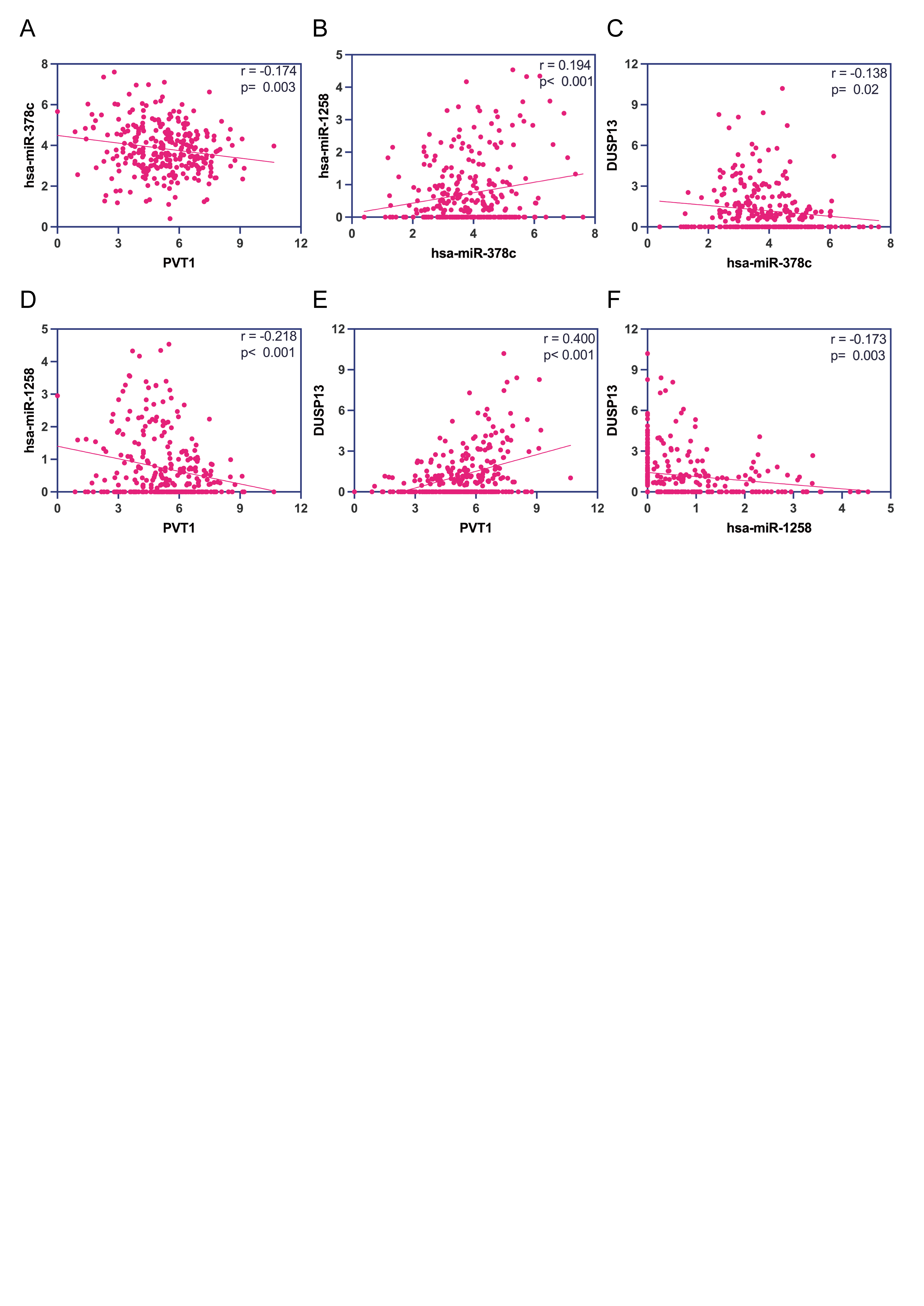

Supplement: Supplementary file 3 — Figure S3. [file CAM4-12-8937-s006.tiff]
