# Supplementary material for: Systemic analysis identifying PVT1/DUSP13 axis for microvascular invasion in hepatocellular carcinoma
Source: Cancer Med. 2022 Dec 16;12(7):8937–55. doi: 10.1002/cam4.5546 (PMC10134337; doi:10.1002/cam4.5546)
Supplement: Supplementary file 4 — Table S1. [file CAM4-12-8937-s005.docx]

| **Supplementary Table S1\| Clinical information for four tissue microarrays with 52 paired HCC and adjacent normal tissues** | | | |
| --- | --- | --- | --- |
| **Overall** | **MVI** | **None** | **p-value** |
|  | 8 (15.4%) | 44 (84.6%) |  |
| Clinicopathologic characteristics | | | |
| Gender |  |  |  |
| Female | 0 (0%) | 7 (15.9%) | 0.516 |
| Male | 8 (100%) | 37 (84.1%) |  |
| Age (mean (SD)) | 56.8 (9.4) | 54.2 (10.5) | 0.780 |
| Tumor size(cm) (mean (SD)) | 7.9 (3.1) | 5.5 (2.4) | 0.023 |
| Pathological Grade (%) |  |  |  |
| II | 6 (75.0) | 26 (59.1) | 0.608 |
| II-III | 2 (25.0) | 15 (34.1) |  |
| III | 0 (0.0) | 3 (6.8) |  |
| HBsAg (%) |  |  |  |
| Negative | 3 (37.5) | 30 (68.2) | 0.208 |
| Positive | 5 (62.5) | 14 (31.8) |  |
| AFP (ng/mL) (mean) | 13943.8 | 4720.7 | 0.157 |
| Cirrhosis (%) |  |  |  |
| No | 3 (37.5) | 15 (34.1) | 1.000 |
| Yes | 5 (62.5) | 29 (65.9) |  |

HCC, Hepatocellular carcinoma; MVI, microvascular invasion. P value <0.05 is considered statistics significance.
