# Supplementary material for: Systemic analysis identifying PVT1/DUSP13 axis for microvascular invasion in hepatocellular carcinoma
Source: Cancer Med. 2022 Dec 16;12(7):8937–55. doi: 10.1002/cam4.5546 (PMC10134337; doi:10.1002/cam4.5546)
Supplement: Supplementary file 5 — Table S2. [file CAM4-12-8937-s004.docx]

| **Supplementary Table S2\| Clinicopathologic characteristics for four tissue microarrays of HCC patients with different vascular invasion groups** | | | |
| --- | --- | --- | --- |
| **Overall** | **MVI** | **None** | **p-value** |
|  | 26 (29.5%) | 62 (70.5%) |  |
| Clinicopathologic characteristics | | | |
| Gender |  |  |  |
| Female | 3 (11.5%) | 10 (16.1%) | 0.822 |
| Male | 23 (88.5%) | 52 (83.9%) |  |
| Age (mean (SD)) | 54.85 (9.86%) | 56.23 (11.49%) | 0.594 |
| Pathological Grade (%) |  |  |  |
| I | 1 (4.0) | 1 (1.6) | 0.041 |
| I-II | 0 (0.0) | 4 (6.5) |  |
| II | 10 (40.0) | 40 (64.5) |  |
| II-III | 5 (20.0) | 10 (16.1) |  |
| III | 9 (36.0) | 7 (11.3) |  |
| AJCC^†^ (%) |  |  |  |
| I | 0 (0.0) | 13 (21.0) | <0.001 |
| II | 1 (3.8) | 37 (59.7) |  |
| III | 17 (65.4) | 11 (17.7) |  |
| IV | 8 (30.8) | 1 (1.6) |  |
| HBsAg (%) |  |  |  |
| Negative | 2 (8.7) | 9 (14.8) | 0.710 |
| Positive | 21 (91.3) | 52 (85.2) |  |
| AFP (ng/mL) (mean) | 5546.10 | 4267.33 | 0.725 |
| Cirrhosis (%) |  |  |  |
| No | 5 (21.7) | 16 (25.8) | 0.918 |
| Yes | 18 (78.3) | 46 (74.2) |  |

**^†^**The eight version of AJCC stage. HCC, Hepatocellular carcinoma; MVI, microvascular invasion. P value <0.05 is considered statistics significance.
