# Supplementary material for: Systemic analysis identifying PVT1/DUSP13 axis for microvascular invasion in hepatocellular carcinoma
Source: Cancer Med. 2022 Dec 16;12(7):8937–55. doi: 10.1002/cam4.5546 (PMC10134337; doi:10.1002/cam4.5546)
Supplement: Supplementary file 7 — Table S4. [file CAM4-12-8937-s003.docx]

**Supplementary Table S3**| **Prediction analysis of transcription factor for PVT1 in HCC**

| **TF Symbol** | **Start** | **End** | **TSS** | **BSD** | **r^†^** | **r^‡^** | **r**^§^ | **r** ^¶^ |
| --- | --- | --- | --- | --- | --- | --- | --- | --- |
| NONO | 127794523 | 127794544 | 127794532 | 127794532 | 0.35*** | -0.38*** | -0.31*** | 0.29*** |
| RBPJ | 127794490 | 127794585 | 127794532 | 127794532 | 0.34*** | -0.32*** | -0.24*** | 0.29*** |
| RFX5 | 127794494 | 127794611 | 127794532 | 127794532 | 0.37*** | -0.26*** | -0.31*** | 0.22*** |
| SAP130 | 127794540 | 127794567 | 127794532 | 127794532 | 0.33*** | -0.40*** | -0.35*** | 0.21*** |
| MAX | 127794547 | 127794570 | 127794532 | 127794532 | 0.35*** | -0.24*** | -0.16** | 0.17*** |
| ZNF792 | 127794511 | 127794610 | 127794532 | 127794532 | 0.27*** | -0.44*** | -0.21*** | 0.21*** |
| HEY1 | 127794514 | 127794610 | 127794532 | 127794532 | 0.34*** | -0.33*** | -0.27*** | 0.23*** |
| MXD4 | 127794555 | 127794580 | 127794532 | 127794532 | 0.34*** | -0.37*** | -0.31*** | 0.28*** |
| MXD3 | 127794518 | 127794625 | 127794532 | 127794532 | 0.41*** | -0.34*** | -0.42*** | 0.32*** |
| PPARG | 127794530 | 127794635 | 127794532 | 127794532 | 0.24*** | -0.24*** | -0.15** | 0.25*** |
| GABPB1 | 127794536 | 127794637 | 127794532 | 127794532 | 0.30*** | -0.30*** | -0.30*** | 0.18*** |
| TFE3 | 127794551 | 127794642 | 127794532 | 127794532 | 0.37*** | -0.33*** | -0.23*** | 0.35*** |
| DMAP1 | 127794585 | 127794610 | 127794532 | 127794532 | 0.26*** | -0.18*** | -0.26*** | 0.27*** |
| DRAP1 | 127794548 | 127794649 | 127794532 | 127794532 | 0.39*** | -0.25*** | -0.30*** | 0.39*** |
| NFIA | 127794569 | 127794636 | 127794532 | 127794532 | -0.19*** | 0.33*** | 0.07 | -0.39*** |
| KLF9 | 127794570 | 127794657 | 127794532 | 127794532 | -0.25*** | 0.36*** | 0.13** | -0.41*** |
| TAF15 | 127794609 | 127794637 | 127794532 | 127794532 | 0.44*** | -0.35*** | -0.36*** | 0.28*** |
| KLF16 | 127794593 | 127794674 | 127794532 | 127794532 | 0.34*** | -0.26*** | -0.17*** | 0.33*** |
| NR2F6 | 127794609 | 127794668 | 127794532 | 127794532 | 0.10* | -0.19*** | -0.21*** | 0.19*** |
| KAT7 | 127794595 | 127794688 | 127794532 | 127794532 | 0.23*** | -0.27*** | -0.31*** | 0.10* |
| ZNF580 | 127794609 | 127794698 | 127794532 | 127794532 | 0.38*** | -0.35*** | -0.30*** | 0.36*** |
| SRSF7 | 127794645 | 127794694 | 127794532 | 127794532 | 0.34*** | -0.37*** | -0.21*** | 0.29*** |
| XRCC5 | 127794726 | 127794770 | 127794532 | 127794532 | 0.19*** | -0.22*** | -0.15** | 0.10* |
| SRSF3 | 127794761 | 127794801 | 127794532 | 127794532 | 0.22*** | -0.34*** | -0.19*** | 0.14** |
| ARID2 | 127795138 | 127795215 | 127794532 | 127794532 | 0.26*** | -0.37*** | -0.29*** | 0.16** |
| KDM1A | 127795172 | 127795255 | 127794532 | 127794532 | 0.34*** | -0.31*** | -0.35*** | 0.31*** |
| MIXL1 | 127795261 | 127795340 | 127794532 | 127794532 | 0.18*** | -0.28*** | -0.02 | 0.24*** |
| ELF3 | 127795341 | 127795420 | 127794532 | 127794532 | 0.17*** | -0.42*** | -0.07 | 0.26*** |

^†^Pearson correlation coefficient between TF and PVT1, ^‡^Pearson correlation coefficient between TF and miR-378c, ^§^Pearson correlation coefficient between TF and miR-1258, ^¶^Pearson correlation coefficient between TF and DUSP13. BSD, Binding site distance; HCC, Hepatocellular carcinoma; ­­TF, Transcription factor; TSS, Transcription start site. P value <0.05 is considered statistics significance. *P<0.05, **P<0.01, ***P<0.001
